# Facile Synthesis of Co Nanoparticles Embedded in N-Doped Carbon Nanotubes/Graphitic Nanosheets as Bifunctional Electrocatalysts for Electrocatalytic Water Splitting

**DOI:** 10.3390/molecules28186709

**Published:** 2023-09-20

**Authors:** Wei Yang, Han Li, Pengzhang Li, Linhua Xie, Yumin Liu, Zhenbao Cao, Chuanjin Tian, Chang-An Wang, Zhipeng Xie

**Affiliations:** 1 School of Mechanical and Electronic Engineering, Jingdezhen Ceramic University, Jingdezhen 333403, China; 2 Institute of New Energy Materials and Devices, School of Materials Science and Engineering, Jingdezhen Ceramic University, Jingdezhen 333403, China; 3 State Key Lab of New Ceramics and Fine Processing, School of Materials Science and Engineering, Tsinghua University, Beijing 100084, China

**Keywords:** HER, OER, Co nanoparticles, bifunctional electrocatalyst, water splitting

## Abstract

Developing robust and cost-effective electrocatalysts to boost hydrogen evolution reactions (HERs) and oxygen evolution reactions (OERs) is crucially important to electrocatalytic water splitting. Herein, bifunctional electrocatalysts, by coupling Co nanoparticles and N-doped carbon nanotubes/graphitic nanosheets (Co@NCNTs/NG), were successfully synthesized via facile high-temperature pyrolysis and evaluated for water splitting. The morphology and particle size of products were influenced by the precursor type of the cobalt source (cobalt oxide or cobalt nitrate). The pyrolysis product prepared using cobalt oxide as a cobalt source (Co@NCNTs/NG-1) exhibited the smaller particle size and higher specific surface area than that of the pyrolysis products prepared using cobalt nitrate as a cobalt source (Co@NCNTs/NG-2). Notably, Co@NCNTs/NG-1 displayed much lower potential −0.222 V vs. RHE for HER and 1.547 V vs. RHE for OER at the benchmark current density of 10 mA cm^−2^ than that of Co@NCNTs/NG-2, which indicates the higher bifunctional catalytic activities of Co@NCNTs/NG-1. The water-splitting device using Co@NCNTs/NG-1 as both an anode and cathode demonstrated a potential of 1.92 V to attain 10 mA cm^−2^ with outstanding stability for 100 h. This work provides a facile pyrolysis strategy to explore highly efficient and stable bifunctional electrocatalysts for water splitting.

## 1. Introduction

Fossil fuel exhaustion and environmental pollution have prompted the development of sustainable and renewable energy technologies. Hydrogen (H_2_) is considered an excellent energy carrier due to its advantages such as cleanliness, high energy density (142 MJ kg^−1^), and sustainability [1]. Electrocatalytic water splitting has received increasing attention as a means to produce green hydrogen (H_2_). Water splitting involves two half-cell reactions of the hydrogen evolution reaction (HER) and the oxygen evolution reaction (OER) [2,3]. The state-of-the-art noble metal OER catalysts (e.g., Pt/C) and noble metal HER catalysts (e.g., RuO_2_ and IrO_2_) have demonstrated superior catalytic activity and the ability to accelerate sluggish kinetics in respective fields. However, the industrial applications of noble metal catalysts have been greatly limited by the scarcity, poor durability, and high cost [4,5]. Given this situation, the exploration of alternative highly stable and cost-effective catalysts with favorable catalytic activities for both OER and HER is vital for the massive commercialization of water splitting [6,7].

Up to now, transition metals and nitrogen containing carbon nanohybrids (M-N-C, M=Ni, Co, Fe, etc.) have been identified as a promising family among various potential candidates due to their cost-effectiveness, structural stability, and high catalytic activities for water splitting [8,9]. Transition metals in M-N-C could improve the electrical conductivity and crystallinity of the carbon matrix during high temperature pyrolysis. Correspondingly, the carbon matrix can inhibit the agglomeration of the transition metal nanoparticles as well as protect transition metal nanoparticles from corrosion in harsh environments during OER and HER processes [10]. Additionally, heteroatoms (e.g., N atoms) doping into the carbon matrix can change exterior electronic structures and improve the exterior hydrophilicity of the carbon matrix and then affect the activity and stability of catalysts [7,11].

Transition metals are effective catalysts for growing carbon nanotubes (CNTs) due to their high carbon solubilities and high diffusion rates at high temperatures [12,13]. In particular, Co is often applied in growing high-quality CNTs [8,14]. Cobalt- and heteroatom--co-doped carbon matrixes have generally been regarded as promising alternatives due to their remarkable catalytic activities in OER and HER. It is noteworthy that nitrogen-doped CNTs with anchored cobalt nanoparticles have demonstrated excellent HER catalytic activity in a wide range of pHs [15]. Moreover, surface adsorption ability toward OOH* intermediates of the carbon matrix could be modified via the metal species and N-doping, thus contributing to the improvement of OER kinetics [16,17].

Transition metal nanoparticles supported on graphene-based catalysts have been prepared through various methods [7]. Generally, the synthesis route has a great impact on the electrocatalytic activity and stability of the as-prepared catalyst. M-N-C catalysts are usually obtained by pyrolyzing the mixtures of transition metal salts (or transition metal oxides) with carbon precursors and/or nitrogen precursors [18]. The high-temperature pyrolysis route is not complicated. However, it is difficult to accurately control the microstructure of the synthesized powder prepared via pyrolysis, which limits the full exposure of electrocatalytic active sites and the further improvement of catalytic activities [19]. The properties and qualities of carbon-based catalysts highly depend on the type of transition metal salt precursor and the relative content of the transition metal salt precursor [4]. The addition of transition metals in pyrolysis precursors contributes to the graphitization of carbon, which significantly affects the stability of the as-prepared catalyst [7,11]. The microstructure of pyrolysis products transform from a graphene-like nanoshell to thread-like carbon with the increase of the relative mass of cobalt nitrate in the precursor, as previously reported [20]. Therefore, the morphology and properties of pyrolysis products are strongly dependent on the type and composition of the cobalt precursor.

In this work, solid-phase precursors using cobalt nitrate (Co(NO_3_)_2_·6H_2_O) or cobalt oxide (Co_3_O_4_) as Co source, glucose as C source, and urea as N source for N doping to prepare cobalt nanoparticles encapsulated in NCNTs and N-doped graphitic nanosheets (NG) by the facile pyrolysis route. The effects of various cobalt sources (Co(NO_3_)_2_·6H_2_O or Co_3_O_4_) on the morphology and electrocatalytic activities of pyrolysis products for HER and OER were systematically studied. The durability for HER and OER of as-prepared catalysts was also carefully evaluated. Anchoring cobalt nanoparticles onto carbon substrates via the high temperature pyrolysis route helps to strengthen the connection between cobalt nanoparticles and the carbon substrate (carbon nanotubes or graphene), which in turn contributes to the improvement of catalytic activities and stability for HER and OER. The water-splitting device was assembled using Co@NCNTs/NG-1 as both the anode and cathode to verify its bifunctional catalytic activities and stability under actual working conditions. All precursors in this work are suitable for large-scale application due to their commercially availability.

## 2. Results and Discussion

**Figure 1:**
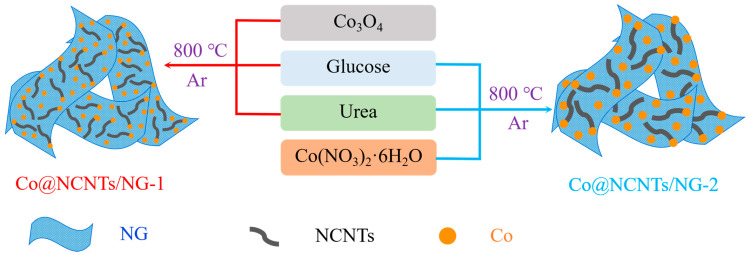
Schematic illustration of the fabrication process of Co@NCNTs/NG-1 and Co@NCNTs/NG-2 electrocatalysts.

A schematic diagram describing the syntheses process of Co@NCNTs/NG-1 and Co@NCNTs/NG-2 is displayed in Figure 1. For Co@NCNTs/NG-1, Co_3_O_4_, glucose, and urea were mixed and then pyrolyzed at 800 °C for 2 h in an Ar atmosphere. The main difference between samples Co@NCNTs/NG-1 and Co@NCNTs/NG-2 is that the Co source of Co@NCNTs/NG-1 is Co_3_O_4_, while the Co source of Co@NCNTs/NG-2 is Co(NO_3_)_2_·6H_2_O.

**Figure 2:**
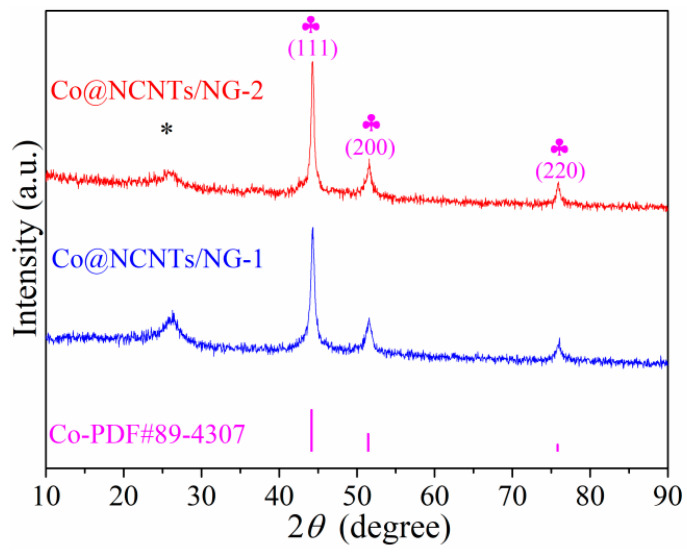
XRD patterns of Co@NCNTs/NG-1 and Co@NCNTs/NG-2 electrocatalysts treated at 800 °C in Ar atmosphere. Asterisks * represent the peak of C.

The metal Co and NCNTs/NG hybrids (denoted as Co@NCNTs/NG-1 and Co@NCNTs/NG-2) were prepared via an uncomplicated, one-step pyrolysis process. The crystal structure of as-prepared samples was determined via the XRD as depicted in Figure 2. The diffraction peaks at 44.2°, 51.5°, and 75.9° can be ascribed to the (111), (200), and (220) planes of the Co phase, respectively. Three peaks of Co phase were consistent with the standard diffraction peaks of metallic Co (PDF#89-4307), indicating the occurrence of metallic Co. Moreover, the broad diffraction peaks centered at 2θ, with a value of about 26.15° for various catalysts, refer to the (002) plane of the graphite 2H (PDF#87-0936), which indicates the existence of graphitic carbon [21]. Therefore, Co@NCNTs/NG-1 and Co@NCNTs/NG-2 were successfully prepared through the facile pyrolysis route.

**Figure 3:**
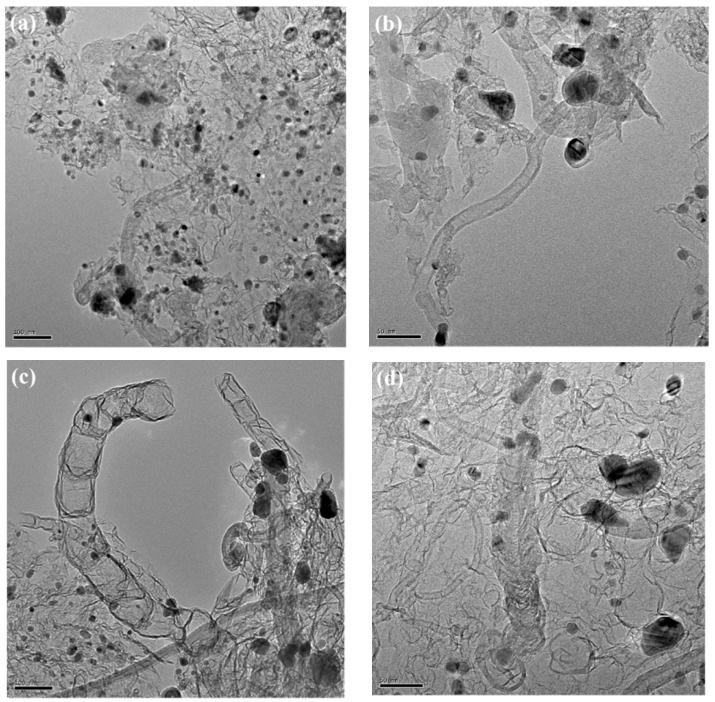
TEM images with different magnifications of (**a**,**b**) Co@NCNTs/NG-1 and (**c**,**d**) Co@NCNTs/NG-2 powders.

The morphology of the Co@NCNTs/NG-1 and Co@NCNTs/NG-2 were further analyzed via SEM and TEM measurements. The metal-catalyzed amorphous carbon conversion to graphene (or carbon nanotubes), during high-temperature pyrolysis, is influenced by the type of cobalt source in the precursor [22]. Co@NCNTs/NG-1 and Co@NCNTs/NG-2 demonstrated porous structures consisting of NCNTs and NG embedded with highly dispersed Co nanoparticles (Figure S1 and Figure 3). NG revealed typical wrinkling characteristics with metallic Co nanoparticles embedded in it [23,24]. It is worth noting that Co nanoparticles of Co@NCNTs/NG-1 were much smaller than that of Co@NCNTs/NG-2, which was beneficial for increasing the specific surface area and expanding the reaction area. Furthermore, the interspersed distribution of NCNTs in Co@NCNTs/NG-1 and Co@NCNTs/NG-2 is conducive to sufficient contact between various phases, which not only facilitates the rapid transfer of electrons and reaction species during the reaction process, but also inhibits the aggregation of Co nanoparticles to improve stability.

**Figure 4:**
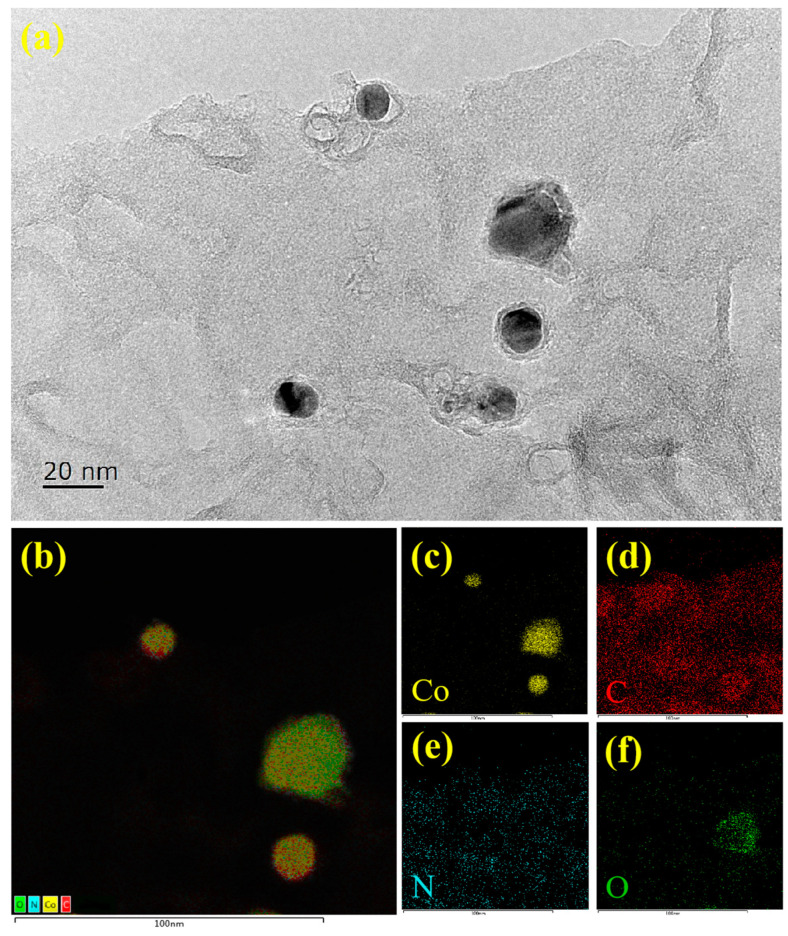
(**a**) TEM images and (**b**–**f**) the corresponding EDX mapping images of Co@NCNTs/NG-1. (A color version of this figure can be viewed online).

Further magnified TEM images display the clear interface between Co nanoparticles and NCNTs (or NG). The energy-dispersive X-ray spectroscopy (EDX) elemental mapping illustrates the existence of Co, C, N, and O in the nanohybrids (Figure 4). Moreover, the C and N elements are evenly distributed in the porous structure (Figure 4d,e), suggesting that the element N had been homogeneously doped into carbon materials (NCNTs or NG) [25,26]. Furthermore, the EDS mapping shows that the Co signal overlap with that of the N signal (Figure 4c,e), which indicatex the existence of Co-N sites [27]. Moreover, the existence of O element was attributed to the partial oxidation of Co nanoparticles.

The content of NCNTs/NG in Co@NCNTs/NG-1 and Co@NCNTs/NG-2 was evaluated via thermal gravimetric analysis (Figure S2). The slow mass loss before 300 °C was mainly attributed to the removal of water and hydroxy groups adsorbed on the surface of hybrids [28]. The weight of various catalysts started to drop above 300 °C, which corresponded to the oxidation of NCNTs or NG [29]. The remaining weight at 800 °C was ascribe to the weight ratio of Co_3_O_4_ derived from the Co@NCNTs/NG-1 or Co@NCNTs/NG-2 [30]. Ultimately, the weight compositions of NCNTs/NG in Co@NCNTs/NG-1 and Co@NCNTs/NG-2 were about 72.84 wt.% and 64.61 wt.%, respectively. The type of cobalt source in the precursor can affect the morphology and carbon content of the final pyrolysis products [31].

**Figure 5:**
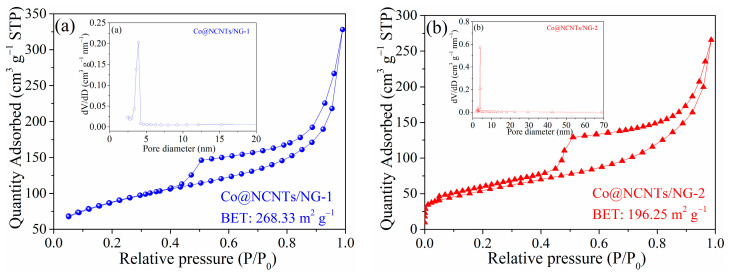
N_2_ adsorption–desorption isotherms and corresponding pore diameter distribution (inset) for (**a**) Co@NCNTs/NG-1 and (**b**) Co@NCNTs/NG-2.

N_2_ adsorption–desorption experiments were carried out to investigate the porosity and specific surface area of various catalysts. The N_2_ adsorption–desorption isotherms of Co@NCNTs/NG-1 and Co@NCNTs/NG-2 in Figure 5 displayed a type IV isotherm with with an H3-type hysteresis loop appearing at P/P_0_ = 0.4–1.0. The pore size distribution of Co@NCNTs/NG-1 and Co@NCNTs/NG-2 was attributed mainly to the mesopore diameters (around 4 nm). According to the results of the Brunauer–Emmett–Teller (BET) analysis, Co@NCNTs/NG-1 (268.33 m^2^ g^−1^) displayed a larger surface area than that of Co@NCNTs/NG-2 (196.25 m^2^ g^−1^), which is conducive to the expansion of the reaction area. Moreover, mesopore channels would facilitate the rapid diffusion and mass transport during the reaction process, thereby speeding up the reaction kinetics [32].

**Figure 6:**
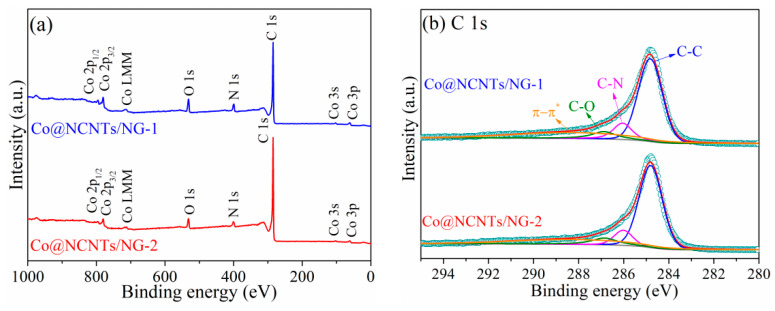

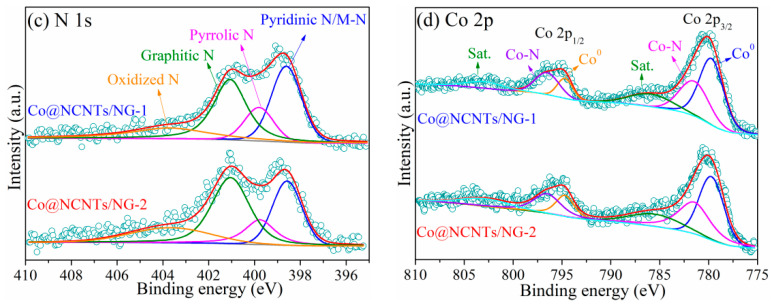
(**a**) XPS survey spectrum and high-resolution spectra of (**b**) C 1s, (**c**) N 1s, and (**d**) Co 2p of Co@NCNTs/NG-1 and Co@NCNTs/NG-2.

The surface chemical composition and valence state of the as-prepared Co@NCNTs/NG-1 and Co@NCNTs/NG-2 were investigated using XPS. As seen in Figure 6a, the typical peaks of Co 2p, N 1s, and C 1s were detected in the XPS survey spectra of Co@NCNTs/NG-1 and Co@NCNTs/NG-2, which indicated the presence of Co, N, and C elements. The high-resolution spectra of C 1s (Figure 6b) were split into C-C (284.8 eV), C-N (286.0 eV), C-O (286.9 eV), and π-π* (288.2 eV) [33]. The presence of a C-N bond verified the successful embedment of N dopants into carbon matrix [34,35]. The N 1s peak (Figure 6c) was split into four peaks, assigned to pyridinic N (398.6 eV), pyrrolic-N (399.8 eV), graphitic-N (401.0 eV) and oxidized-N (403.7 eV), respectively. It should be noted that the peak located at 398.6 eV also includes the contribution of the biding to Co-N, which is due to the similar binding energy of pyridinic N and Co-N [36]. The dominant pyridinic-N and graphitic-N within as-prepared catalysts could improve the charge transfer, thus leading to the enhanced catalytic activities [34,37]. Moreover, the Co 2p peaks (Figure 6d) were deconvoluted into the Co-N (781.5 eV for Co 2p_3/2_ and 796.5 eV for Co 2p_1/2_) and metallic Co^0^ species (779.7 eV for Co 2p_3/2_ and 794.7 eV for Co 2p_1/2_), which were further confirmed by the weak Co 2p satellite peaks (785.5 eV for Co 2p_3/2_ and 802.9 eV for Co 2p_1/2_) [38,39]. The presence of Co-N bonds contributes to the improvement of OER and HER catalytic activities, which has been confirmed by previous studies [29]. Metallic Co nanoparticles encapsulated on NCNTs and NG can provide facile routes for electron transfer.

**Figure 7:**
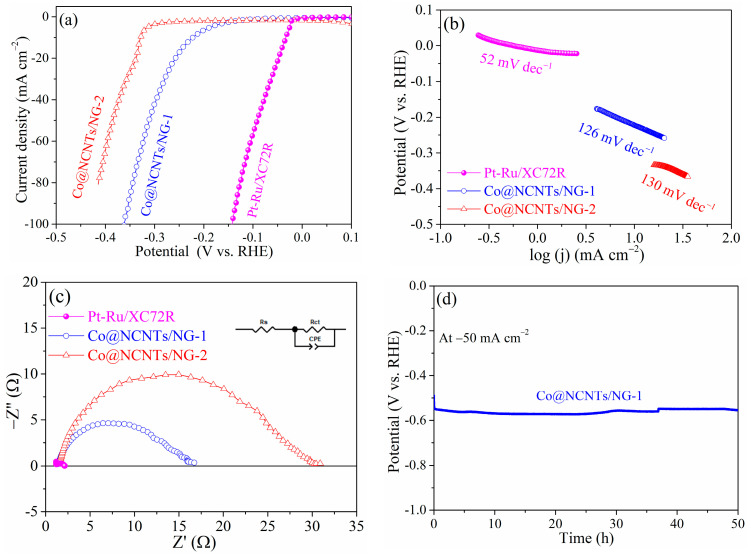
(**a**) HER polarization curves of various electrocatalysts and corresponding (**b**) Tafel plots; (**c**) EIS spectra tested at −0.176 V vs. RHE; (**d**) the chronopotentiometric curve of the Co@NCNTs/NG-1 electrode measured at 50 mA cm^−2^.

To assess the catalytic properties for HER, the as-prepared Co@NCNTs/NG-1, Co@NCNTs/NG-2, and the commercial Pt-Ru/XC72R were characterized in 1. 0 M KOH using a typical three-electrode configuration at a scan rate of 10 mV s^−1^. It is worth noting that the observed potentials at the benchmark current density of 10 mA cm^−2^ were −0.222 V vs. RHE, −0.328 V vs. RHE, and −0.033 V vs. RHE for Co@NCNTs/NG-1, Co@NCNTs/NG-2 and Pt-Ru/XC72R (Figure 7a), respectively. Apart from the commercial Pt-Ru/XC72R with excellent HER catalytic activity, Co@NCNTs/NG-1 demonstrated higher HER catalytic activity than that of Co@NCNTs/NG-2. The overpotential at 10 mA cm^−2^ (η_10_) and Tafel slope of Co@NCNTs/NG-1 and Co@NCNTs/NG-2 for HER were compared with those of previously reported Co-based catalysts as shown in Table S1. The η_10_ of Co@NCNTs/NG-1 was 222 mV vs. RHE, which was lower than that of Co@NCNTs/NG-2 (328 mV vs. RHE), Co-PNCNFs (249 mV vs. RHE) [40], Co/CNT (298 mV vs. RHE) [41], Co NPs@N-CNTs (370 mV vs. RHE) [15] and comparable to the Co@NG (220 mV vs. RHE) [42], Co@N-C (210 mV vs. RHE) [43], Co/Co_8_S_9_/CNT (200 mV vs. RHE) [41], Co/CNFs (1000) (190 mV vs. RHE) [28], respectively.

Indeed, the rapid HER reaction kinetics of Co@NCNTs/NG-1 was further confirmed through its small Tafel slope (126 mV dec^−1^), which was slightly lower than that of the Co@NCNTs/NG-2 (130 mV dec^−1^) (Figure 7b). The Tafel slope of Co@NCNTs/NG-1 (126 mV dec^−1^) was comparable to reported Co-based composite catalysts, such as Co/CNT (152 mV dec^−1^) [41], Co@NG (112 mV dec^−1^) [42], Co@N-C (108 mV dec^−1^) [43]. As expected, the commercial Pt-Ru/XC72R displayed the smallest Tafel slope value (52 mV dec^−1^), which is similar to previous reports on Pt/C [41,44]. The relatively small Tafel slope value of Co@NCNTs/NG-1 indicated the higher intrinsic HER reaction rate and faster catalytic reaction kinetics.

Moreover, EIS spectra at the potential of −0.176 V vs. RHE was performed to study the charge transfer impedance (R_ct_) in the HER process. EIS data were fitted to the equivalent circuit of R_s_(R_ct_, C_dl_) (inset of Figure 7c, Table S2). R_s_, CPE, and R_ct_ represented the resistance of the electrolyte in series, the constant-phase element, as well as the charge transfer resistance, respectively [45]. Co@NCNTs/NG-1 displayed a lower R_s_ value (1.35 Ω) than that of Co@NCNTs/NG-2 (1.48 Ω), indicating the higher electron conductivity of Co@NCNTs/NG-1. The R_ct_ value was 13.1 Ω, 26.4 Ω, and 1.05 Ω for Co@NCNTs/NG-1, Co@NCNTs/NG-2, and the commercial Pt-Ru/XC72R, respectively. The lower charge transfer resistance R_ct_ of Co@NCNTs/NG-1 than that of Co@NCNTs/NG-2 indicates more favorable charge transfer kinetics, which are conducive to the improvement of the HER catalytic activity [46].

The durability and stability of Co@NCNTs/NG-1 in the HER process was evaluated via chronopotentiometric measurement without iR compensation. Co@NCNTs/NG-1 demonstrated a stable potential for 50 h at the constant current density of 50 mA cm^−2^ (Figure 7d), suggesting its outstanding stability under the alkaline HER environment. In fact, the interaction between Co nanoparticles and NCNTs (or NG) played a crucial role in enhanced OER and/or HER electrocatalytic activities. The tight anchoring of cobalt nanoparticles on NCNTs (or NG) was beneficial for inhibiting the agglomeration of cobalt nanoparticles during the pyrolysis process. Furthermore, the durability of Co@NCNTs/NG-1 and Co@NCNTs/NG-2 for OER and/or HER could be improved due to the strong interaction between Co nanoparticles and NCNTs (or NG) [47].

**Figure 8:**
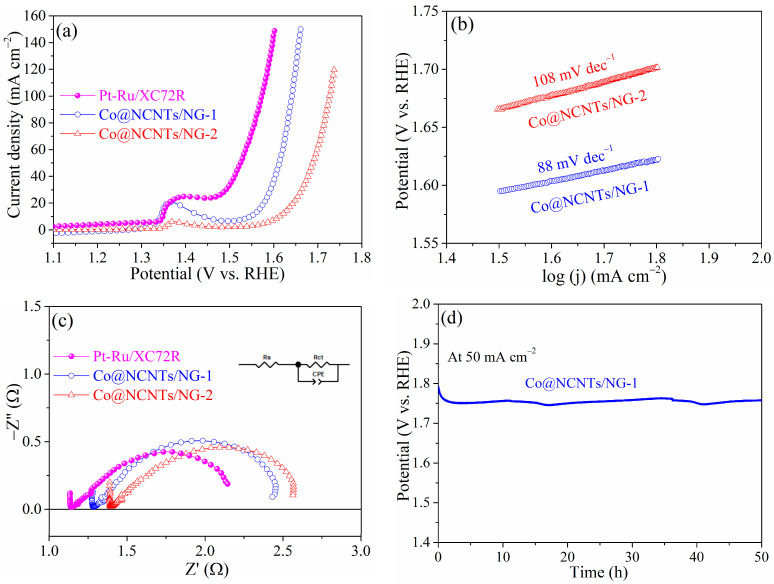
(**a**) OER polarization curves of Co@NCNTs/NG-1, Co@NCNTs/NG-2, and Pt-Ru/XC72R; (**b**) Tafel plots of Co@NCNTs/NG-1 and Co@NCNTs/NG-2 derived from the OER polarization curves in (**a**); (**c**) EIS spectra tested at 1.624 V vs. RHE; (**d**) the chronopotentiometric curve of the Co@NCNTs/NG-1 electrode measured at 50 mA cm^−2^.

In addition to the HER activity, the OER activity also plays a crucial role in highly efficient water splitting. The OER catalytic activities of various catalysts were also investigated in a typical three-electrode system. As expected, Pt-Ru/XC72R displayed the best OER catalytic activity with the lowest potential according to the LSV curves for OER (Figure 8a). Notably, Co@NCNTs/NG-1 displayed much lower potential (1.547 V vs. RHE) at the benchmark current density of 10 mA cm^−2^ than that of Co@NCNTs/NG-2 (1.613 V vs. RHE), which indicates higher OER catalytic activities of Co@NCNTs/NG-1. Moreover, kinetic parameters were also evaluated via the Tafel slope in Figure 8b. Co@NCNTs/NG-1 displayed a much lower Tafel slope (88 mV dec^−1^) than that of Co@NCNTs/NG-2 (108 mV dec^−1^), suggesting its faster OER kinetics [48]. It is noteworthy that the η10 and Tafel slope of Co@NCNTs/NG-1 and Co@NCNTs/NG-2 for OER were close to or even smaller than that of the Co-based catalysts previously reported (Table S3).

Electrode kinetics for OER were also evaluated via EIS spectra at 1.624 V vs. RHE. EIS spectra were fitted to an equivalent circuit (Figure 8c, Table S4). Co@NCNTs/NG-1 exhibited an R_ct_ (1.23 Ω) which was smaller than Co@NCNTs/NG-2 (1.31 Ω) and close to Pt-Ru/XC72R (1.16 Ω), which indicates a better intrinsic charge-transfer property for OER kinetics [49]. The electrochemical active surface area (ECSA) of Co@NCNTs/NG-1 was evaluated by measuring the electrochemical double-layer capacitance (C_dl_) by scanning CV in the non-Faraday region at different scan rates (Figure S3a). The C_dl_ value of Co@NCNTs/NG-1 was 0.33 mF cm^−2^ (Figure S3b), suggesting the greater exposure of electrochemical surface, which can provide more active sites for both HER and OER processes.

Moreover, the long-term OER durability of Co@NCNTs/NG-1 was evaluated via the chronopotentiometric curve without iR compensation. The potential at the constant current density of 50 mA cm^−2^ of Co@NCNTs/NG-1 remained stable for 50 h (Figure 8d), revealing that it is highly durable in the alkaline OER environment.

**Figure 9:**
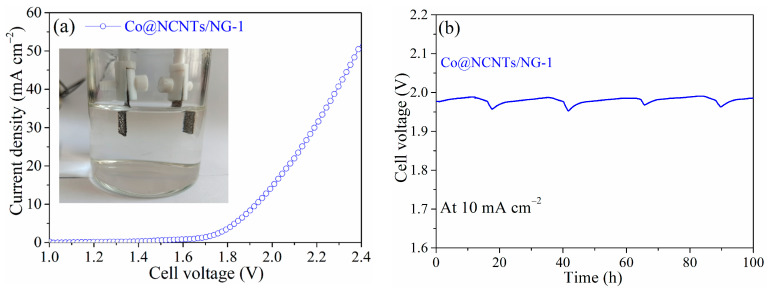
(**a**) Polarization curves of Co@NCNTs/NG-1 for overall water splitting (inset: the digital photograph of the water-splitting device in a two-electrode configuration) and (**b**) durability test carried out at 10 mA cm^−2^ for 100 h.

Encouraged by the high catalytic activity and durability of Co@NCNTs/NG-1 for both HER and OER, the water-splitting device was assembled using Co@NCNTs/NG-1 as both the anode and cathode (Co@NCNTs/NG-1‖ Co@NCNTs/NG-1) as shown in (inset of Figure 9a). Notably, the water-splitting device with Co@NCNTs/NG-1 required a voltage of 1.92 V at 10 mA cm^−2^ and a voltage of 2.38 V at 50 mA cm^−2^ without iR compensation (Figure 9a), indicating its satisfactory activities for overall water splitting. The satisfactory electrocatalytic activities of Co@NCNTs/NG-1 and Co@NCNTs/NG-2 for both OER and HER were mainly attributed to the tightly anchoring of Co nanoparticles and NCNTs (or NG), Co-N and pyridinic N sites as well as the porous structure constructed by NCNTs and NG [31,47]. Furthermore, the long-term stability test was also carried out to assess the stability of Co@NCNTs/NG-1 in an actual water splitting environment. The cell voltage at 10 mA cm^−2^ was almost stable after 100 h (Figure 9b), which further confirmed the promising bifunctional catalytic activities and outstanding stability of Co@NCNTs/NG-1.

## 3. Experimental Details

### 3.1. Catalyst Synthesis

All reagents in this work were analytical-grade. All catalysts were prepared via an uncomplicated pyrolysis route. Briefly, 1.67 mmol of Co_3_O_4_, 6 mmol of glucose and 0.4 mol urea were mixed and ground for 1 h as the precursor. The precursor was calcined at 800 °C for 2 h under the flow of Ar gas to obtain the black power (denoted as Co@NCNTs/NG-1). For comparison, 5 mmol of Co(NO_3_)_2_·6H_2_O, 6 mmol of glucose and 0.4 mol urea were also mixed and ground for 1 h as the precursor and then subjected to pyrolysis at 800 °C for 2 h (denoted as Co@NCNTs/NG-2).

### 3.2. Material Characterization

The phase structures of Co@NCNTs/NG-1 and Co@NCNTs/NG-2 catalysts were characterized via X-ray diffraction (XRD, Bruker D8, Karlsruhe, Germany) with Cu-Kα radiation (λ = 0.15418 nm). The morphologies and elements distribution of as-prepared catalysts were evaluated via a field emission scanning electron microscope (SEM, Zeiss Sigma 500, Tokyo, Japan) and transmission electron microscopy (TEM, FEI Talos F200S, Hillsboro, OR, USA). The surface chemical composition and valence state of as-prepared catalysts were evaluated via X-ray photoelectron spectroscopy (XPS, Thermo ESCALAB 250XI, Waltham, MA, USA). The binding energy of all XPS spectra was calibrated with the C 1s peak (284.6 eV). The specific surface area of catalysts was assessed via the Brunauer–Emmett–Teller (BET) method. The weight percentage of NCNTs/ NG in as-prepared samples was determined via the thermogravimetric analyzer (TGA, TA SDT650, New York, NY, USA) from room temperature to 800 °C with a heating rate of 5 °C min^−1^ under the air flow.

### 3.3. Electrochemical Measurements

The nickel foam (nickel content of 99.9 wt%) with a thickness of 1.0 mm was purchased from Taiyuan Lizhiyuan Co., Taiyuan, China. The nickel foam was cut into pieces (geometric area: 1 cm × 2 cm), which were sonicated in 1 M HCl aqueous solution for 30 min and further washed with deionized water and ethanol. The catalyst ink was obtained by dispersing 10 mg of the as-prepared catalyst and 10 mg of conductive carbon (Super P Li, TIMCAL, Bodio, Switzerland) into the mixture of 70 μL of Nafion solution (5 wt%), 530 μL of ethanol and 400 μL of deionized water. The ink was sonicated for 1 h to ensure uniform dispersion. Subsequently, 100 μL of the catalyst ink was carefully dropped onto the pre-cleaned nickel foam substrate (geometric area: 1 cm × 1 cm) with a nominal catalyst mass loading of about 0.43 mg cm^−2^ (Figure S4). For comparison, the commercial Pt-Ru/XC72R (HISPEC10000, Pt 40 wt% and Ru 20 wt%, Johnson Matthey, London, UK) electrodes were also prepared using a similar procedure. The prepared electrode was applied to evaluate OER and HER activities of as-prepared catalysts as well as its properties of overall water splitting.

Electrochemical tests including linear sweep voltammetry (LSV), cyclic voltammery (CV), electrochemical impedance spectroscopy (EIS), and the chronopotentiometric curve were performed using a DH7000 electrochemical workstation (Jiangsu Dong Hua, Jingjiang, China) with a typical three-electrode configuration at room temperature. The three-electrode configuration was composed of a working electrode based on nickel foam coating via catalysts, an Hg/HgO (1 M KOH electrolyte) reference electrode, and a graphite rod as the counter electrode. 1 M KOH served as the electrolyte. All LSV curves in this work were recorded at a scan rate of 10 mV s^−1^ with iR compensation. EIS was recorded in a frequency range between 100 kHz and 0.1 Hz with an AC amplitude of 10 mV. All the potentials gauged in the Hg/HgO (1 M KOH electrolyte) reference electrode were converted to the reversible hydrogen electrode (RHE) according the following equation: *E*_RHE_ = *E*_Hg/HgO_ + 0.098 + 0.059 × pH [50,51]. The overpotential (η) of the OER was acquired with the equation, η = *E*_RHE_ − 1.23 V [52]. The current density was normalized according to the geometric area [53]. The water splitting measurements were conducted in a two-electrode system with Co@NCNTs/NG-1 as anode and cathode.

## 4. Conclusions

In this work, Co@NCNTs/NG-1 and Co@NCNTs/NG-2 were successfully prepared via a facile pyrolysis strategy using cobalt oxide and cobalt nitrate as cobalt sources, respectively. Co@NCNTs/NG-1 and Co@NCNTs/NG-2 revealed porous structures consisting of NCNTs and NG embedded with highly dispersed Co nanoparticles. Notably, Co@NCNTs/NG-1 displayed much lower potential −0.222 V vs. RHE for HER and 1.547 V vs. RHE for OER at 10 mA cm^−2^ than that of Co@NCNTs/NG-2, which indicates the higher HER and OER catalytic activities of Co@NCNTs/NG-1. The tight anchoring of cobalt nanoparticles on NCNTs (or NG) was beneficial for inhibiting the agglomeration and activity decline of cobalt nanoparticles, thereby improving the catalytic activity and stability of the as-prepared catalyst. Based on the results, this work provides a facile pyrolysis strategy for designing highly efficient cobalt-based bifunctional electrocatalysts for water splitting.

## Data Availability

Not applicable.

## Supplementary Materials

The following supporting information can be downloaded at https://www.mdpi.com/article/10.3390/molecules28186709/s1, Figure S1: SEM images of Co@NCNTs/NG-1 and Co@NCNTs/NG-2 powders; Figure S2: TG curves of Co@NCNTs/NG-1 and Co@NCNTs/NG-2 in air; Figure S3: (a) CV curves for Co@NCNTs/NG-1 at various scan rates, (b) The linear fitting of capacitive current densities at 0.924 V vs. RHE; Figure S4: The digital photograph of the prepared Co@NCNTs/NG-1 electrode; Table S1: Comparison of HER of as-synthesized catalysts with recently reported Co-based catalyst; Table S2: The values of the equivalent circuit elements resulting from fitting the EIS data tested at −0.176 V vs. RHE of Co@NCNTs/NG-1, Co@NCNTs/NG-2 and Pt-Ru/XC72R; Table S3: Comparison of OER of as-synthesized catalysts with recently reported Co-based catalyst; Table S4: The values of the equivalent circuit elements resulting from fitting the EIS data tested at 1.624 V vs. RHE of Co@NCNTs/NG-1, Co@NCNTs/NG-2 and Pt-Ru/XC72R. References [54,55,56,57,58,59] are cited in the supplementary materials.
