# Supplementary material for: Facile Synthesis of Co Nanoparticles Embedded in N-Doped Carbon Nanotubes/Graphitic Nanosheets as Bifunctional Electrocatalysts for Electrocatalytic Water Splitting
Source: Molecules. 2023 Sep 20;28(18):6709. doi: 10.3390/molecules28186709 (PMC10535278; doi:10.3390/molecules28186709)
Supplement: Supplementary file 1 [file molecules-28-06709-s001.zip › molecules-2591226-supplementary.pdf]

Supporting Information

Facile synthesis of Co nanoparticles embedded in N-doped carbon nanotubes/graphitic nanosheets as bifunctional electrocatalysts for electrocatalytic water splitting

Wei Yang[1,*], Han Li[2], Pengzhang Li[2,*], Linhua Xie[2], Yumin Liu[2], Zhenbao Cao[2], Chuanjin Tian[2], Chang-An Wang[2,3], Zhipeng Xie[2,3]

[1]School of Mechanical and Electronic Engineering, Jingdezhen Ceramic University, Jingdezhen 333403, China

[2]Institute of New Energy Materials and Devices, School of Materials Science and Engineering, Jingdezhen Ceramic University, Jingdezhen 333403, China

[3]State Key Lab of New Ceramics and Fine Processing, School of Materials Science and Engineering, Tsinghua University, Beijing 100084, China

[*] Correspondence: 13s011016@hit.edu.cn (W.Y.); 15b911005@hit.edu.cn (P.L.).

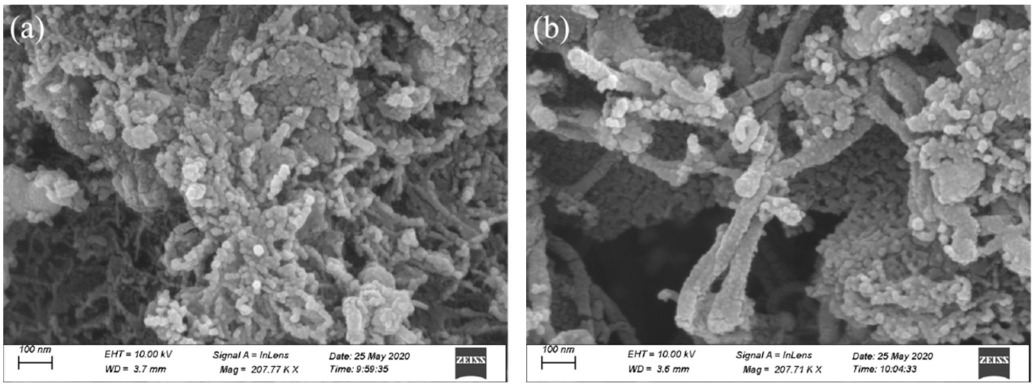

Figure S1. SEM images of (a) Co@NCNTs/NG-1 and (b) Co@NCNTs/NG-2 powders.

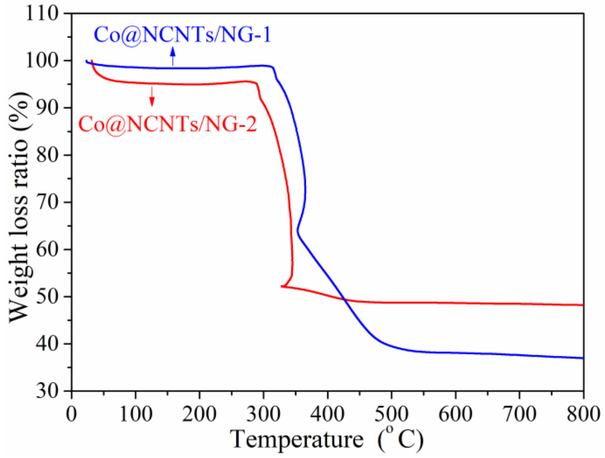

Figure S2. TG curves of Co@NCNTs/NG-1 and Co@NCNTs/NG-2 in air.

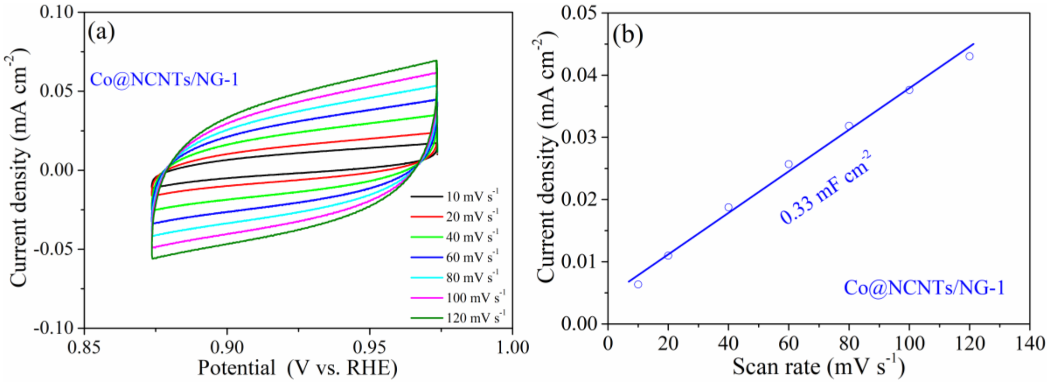

Figure S3. (a) CV curves for Co@NCNTs/NG-1 at various scan rates; (b) The linear fitting of capacitive current densities at 0.924 V vs. RHE.

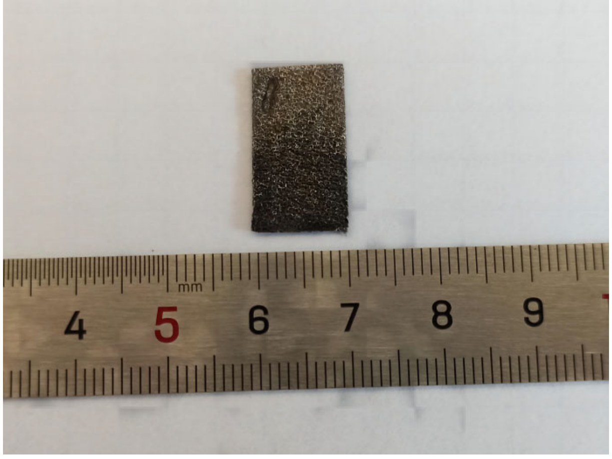

Figure S4. The digital photograph of the prepared Co@NCNTs/NG-1 electrode.

Table S1. Comparison of HER of as-synthesized catalysts with recently reported Co-based catalyst.

Catalysts	η_{10} (mV vs. RHE)	Tafel slope (mV dec^{-1})	Electrolyte	Reference
Co@NCNTs/NG-1	222	126	1 M KOH	This work
Co@NCNTs/NG-2	328	130	1 M KOH	This work
Pt-Ru/XC72R	33	52	1 M KOH	This work
Co/CNFs (1000)	190	66	1 M KOH	[1]
Co/Co_8S_9/CNT	200	90	1 M KOH	[2]
$Co_{0.5}$-NCNT@NHC	208	137	1 M KOH	[3]
Co@N-C	210	108	1 M KOH	[4]
Co@NG	220	112	1 M KOH	[5]
Co-PNCNFs	249	92	1 M KOH	[6]
CoS_2QDs@rGO	298	78	1 M KOH	[7]
Co/CNT	298	152	1 M KOH	[4]
Co NPs@N-CNTs	370	Not given	1 M KOH	[8]

Table S2. The values of the equivalent circuit elements resulting from fitting the EIS data tested at −0.176 V vs. RHE of Co@NCNTs/NG-1, Co@NCNTs/NG-2 and Pt-Ru/XC72R.

Element	Co@NCNTs/NG-1	Co@NCNTs/NG-2	Pt-Ru/XC72R
R_s (Ω)	1.35	1.48	1.09
R_{ct} (Ω)	13.1	26.4	1.05

Table S3. Comparison of OER of as-synthesized catalysts with recently reported Co-based catalyst.

Catalysts	η_{10} (mV vs. RHE)	Tafel slope (mV dec^{-1})	Electrolyte	Reference
Co@NCNTs/NG-1	317	88	1 M KOH	This work
Co@NCNTs/NG-2	383	108	1 M KOH	This work
Co/Co_8S_9/CNT	260	79	1 M KOH	[2]
Ni_3S_2/MoS_2	303	70.3	1 M KOH	[9]
Co/N-CNTs	310	66	1 M KOH	[10]
Co/CNFs (1000)	320	79	1 M KOH	[1]
Co_2P@Co/N-C	320	48.8	1 M KOH	[11]
Co/PNG	335	63	1 M KOH	[12]
Co-P/Co-N-C/NPC	374	92	1 M KOH	[13]
Co@N-C	400	Not given	1 M KOH	[4]

Table S4. The values of the equivalent circuit elements resulting from fitting the EIS data tested at 1.624 V vs. RHE of Co@NCNTs/NG-1, Co@NCNTs/NG-2 and Pt-Ru/XC72R.

Element	Co@NCNTs/NG-1	Co@NCNTs/NG-2	Pt-Ru/XC72R
R_s (Ω)	1.33	1.43	1.17
R_{ct} (Ω)	1.23	1.31	1.16

References:

[1] Yang Z, Zhao C, Qu Y, Zhou H, Zhou F, Wang J, Wu Y, Li Y. Trifunctional Self-Supporting Cobalt-Embedded Carbon Nanotube Films for ORR, OER, and HER Triggered by Solid Diffusion from Bulk Metal. Adv Mater 2019, 31(12): e1808043.

[2] Ashok A, Kumar A, Ponraj J, Mansour SA. Development of Co/Co_9S_8 metallic nanowire anchored on N-doped CNTs through the pyrolysis of melamine for overall water splitting. Electrochim Acta 2021, 368: 137642.

[3] Zhuang Y, Cheng H, Meng C, Chen B, Zhou H. Self-catalyzed Co, N-doped carbon nanotubes-grafted hollow carbon polyhedrons as efficient trifunctional electrocatalysts for zinc-air batteries and self-powered overall water splitting. J Colloid Interface Sci 2023, 643: 162-173.

[4] Wang J, Gao D, Wang G, Miao S, Wu H, Li J, Bao X. Cobalt nanoparticles

encapsulated in nitrogen-doped carbon as a bifunctional catalyst for water electrolysis. J Mater Chem A 2014, 2(47): 20067-20074.
[5] Zeng M, Liu Y, Zhao F, Nie K, Han N, Wang X, Huang W, Song X, Zhong J, Li Y. Metallic Cobalt Nanoparticles Encapsulated in Nitrogen-Enriched Graphene Shells: Its Bifunctional Electrocatalysis and Application in Zinc-Air Batteries. Adv Funct Mater 2016, 26(24): 4397-4404.
[6] Zhao Y, Zhang J, Li K, Ao Z, Wang C, Liu H, Sun K, Wang G. Electrospun cobalt embedded porous nitrogen doped carbon nanofibers as an efficient catalyst for water splitting. J Mater Chem A 2016, 4(33): 12818-12824.
[7] Jiang J, Sun R, Huang X, Cong H, Tang J, Xu W, Li M, Chen Y, Wang Y, Han S, Lin H. CoS2 quantum dots modified by ZIF-67 and anchored on reduced graphene oxide as an efficient catalyst for hydrogen evolution reaction. Chem Eng J 2022, 430: 132634.
[8] Zou X, Huang X, Goswami A, Silva R, Sathe BR, Mikmekova E, Asefa T. Cobalt-embedded nitrogen-rich carbon nanotubes efficiently catalyze hydrogen evolution reaction at all pH values. Angewandte Chemie 2014, 53(17): 4372-4376.
[9] Kim S, Min K, Kim H, Yoo R, Shim SE, Lim D, Baeck S-H. Bimetallic-metal organic framework-derived Ni_3S_2/MoS_2 hollow spheres as bifunctional electrocatalyst for highly efficient and stable overall water splitting. Int J Hydrogen Energy 2022, 47(13): 8165-8176.
[10] Han H, Paik JW, Ham M, Kim KM, Park JK, Jeong YK. Atomic Layer Deposition-Assisted Fabrication of Co-Nanoparticle/N-Doped Carbon Nanotube Hybrids as Efficient Electrocatalysts for the Oxygen Evolution Reaction. Small 2020, 16(33): e2002427.
[11] Zhu C, Fu S, Xu BZ, Song J, Shi Q, Engelhard MH, Li X, Beckman SP, Sun J, Du D, Lin Y. Sugar Blowing-Induced Porous Cobalt Phosphide/Nitrogen-Doped Carbon Nanostructures with Enhanced Electrochemical Oxidation Performance toward Water and Other Small Molecules. Small 2017, 13(33): 1700796.
[12] Yang G, Liu J, Zhou M, Bai J, Bo X. Fast and Facile Room-Temperature Synthesis of MOF-Derived Co Nanoparticle/Nitrogen-Doped Porous Graphene in Air Atmosphere for Overall Water Splitting. ACS Sustainable Chem Eng 2020, 8(32): 11947-11955.
[13] Wang S, Jang H, Wang J, Wu Z, Liu X, Cho J. Cobalt-Tannin-Framework-Derived Amorphous Co-P/Co-N-C on N, P Co-

Doped Porous Carbon with Abundant Active Moieties for Efficient Oxygen Reactions and Water Splitting. ChemSusChem 2019, 12(4): 830-838.
